# Factors Associated With Measles Transmission in the United States During the Postelimination Era

**DOI:** 10.1001/jamapediatrics.2019.4357

**Published:** 2019-11-18

**Authors:** Paul A. Gastañaduy, Sebastian Funk, Benjamin A. Lopman, Paul A. Rota, Manoj Gambhir, Bryan Grenfell, Prabasaj Paul

**Affiliations:** 1 Division of Viral Diseases, National Center for Immunizations and Respiratory Diseases, Centers for Disease Control and Prevention, Atlanta, Georgia; 2 Centre for the Mathematical Modelling of Infectious Diseases, London School of Hygiene and Tropical Medicine, London, United Kingdom; 3 Rollins School of Public Health, Emory University, Atlanta, Georgia; 4 Epidemiological Modelling Unit, Monash University, Melbourne, Victoria, Australia; 5 Health Modelling and Analytics Team, IBM Research Australia, Melbourne, Victoria, Australia; 6 Department of Ecology and Evolutionary Biology, Princeton University, Princeton, New Jersey; 7 Division of Healthcare Quality Promotion, Centers for Disease Control and Prevention, Atlanta, Georgia

## Abstract

**Importance:**

Measles cases and outbreaks continue to occur in the United States after the introduction of measles from endemic settings.

**Objective:**

To discern the factors associated with measles transmission in the United States after measles had been eliminated.

**Design, Setting, and Participants:**

This cross-sectional study was conducted from January 1, 2001, to December 31, 2017, in the United States among US residents and international visitors with confirmed measles. A maximum likelihood algorithm that uses the observed dates of rash onset and the known distribution of the serial interval (time between symptom onset in related consecutive cases) was applied to outbreak notification data to estimate the effective reproduction number (*R*), or the mean number of new infections generated per case. Transmissibility was assessed by comparing *R* based on the characteristics of primary and secondary cases of measles.

**Exposures:**

Measles virus.

**Main Outcomes and Measures:**

Effective reproduction number (*R*), or the mean number of successful transmission events per case of measles (ie, the mean number of persons to whom each patient with measles spreads measles).

**Results:**

A total of 2218 individuals with confirmed measles cases (1025 female, 1176 male, and 17 sex not reported; median age, 15 years [range, 0-89 years]) reported from 2001 to 2017 were evaluated. Among patients who received no doses of measles vaccine, *R* was 0.76 (95% CI, 0.71-0.81); among patients who received 1 dose of measles vaccine, *R* was 0.17 (95% CI, 0.11-0.26); among patients who received 2 doses or more of measles vaccine, *R* was 0.27 (95% CI, 0.17-0.39); and among patients with unknown vaccination status, *R* was 0.52 (95% CI, 0.44-0.60). Among patients born before 1957, *R* was 0.35 (95% CI, 0.20-0.58), and among those born on or after 1957, *R* was 0.64 (95% CI, 0.61-0.68). *R* was higher when primary and secondary cases of measles were patients aged 5 to 17 years (0.36 [95% CI, 0.31-0.42]) compared with assortative transmission in other age groups (<1 year, 0.14 [95% CI, 0.10-0.20]; 1-4 years, 0.25 [95% CI, 0.20-0.30]; 18-29 years, 0.19 [95% CI, 0.15-0.24]; 30-49 years, 0.15 [95% CI, 0.11-0.20]; ≥50 years, 0.04 [95% CI, 0.01-0.10]).

**Conclusions and Relevance:**

The findings of this study support having high targets for 2-dose measles vaccine coverage, particularly among school-aged children in the United States.

## Key Points

**Question:**

What are the factors causing the transmission of measles in long-standing measles control programs?

**Findings:**

This cross-sectional study found that lack of vaccination and birth on or after 1957, as well assortative transmission by age (particularly among school-aged children), are the primary factors associated with measles transmission in the United States. Although current measles vaccines are known to be highly effective in decreasing susceptibility to measles, these analyses shed light on the degree by which vaccination also limits measles transmission.

**Meaning:**

The findings underscore the importance of maintaining homogenous, high, 2-dose measles vaccine coverage, especially among school-aged children, to sustain elimination of measles in the United States.

## Teaser

This cross-sectional study examines the factors associated with measles transmission in the United States after measles had been eliminated.

## Introduction

Global coverage with the first dose of a measles vaccine has plateaued at approximately 85% since 2010, increases in measles incidence have been noted in 5 of the 6 World Health Organization Regions since 2016, and at least 1 country in the Americas, Venezuela, has reestablished endemic measles virus transmission.^1^ The decelerating progress in global elimination efforts implies that measles will remain endemic in many parts of the world and that the virus will continue to test immunity levels in elimination settings for the foreseeable future. Sizeable outbreaks have recently occurred in several US states (eg, New York, Washington, and New Jersey)^2^ and in other countries (eg, Canada, Vietnam, and the Philippines), pointing to heterogeneity in vaccination coverage.

Factors other than lack of vaccination might contribute to measles virus transmission in settings with mature control programs. First, as with other respiratory illnesses, measles transmission is affected by contact patterns, particularly mixing within and between age groups.^3^ Second, intense contact and high population density (eg, in schools and metropolitan areas) have been associated with an increased risk for measles outbreaks.^4,5^ Third, studies have shown reduced antibody responses^6^ and a higher risk for measles^7^ when the first dose of the measles vaccine is administered at 12 to 14 months of age compared with when the vaccine is given at 15 months of age or older. Fourth, in the absence of boosting from wild-type disease, vaccine-induced antibody titers are known to decline over time,^8^ and vaccinated persons are potentially susceptible to infection and disease as a result of waning immunity.^9^ The ability of vaccine nonresponders and of individuals with waning immunity to transmit measles is poorly understood.

A better understanding of the factors affecting measles virus transmission could help improve the allocation of public health resources for measles prevention and control in elimination and near-elimination settings. We aimed to discern factors associated with measles virus transmission in the United States after elimination.

## Methods

Measles is nationally notifiable in the United States.^10,11^ Cases are reported by health care professionals and clinical laboratories, investigated by local and state health departments, classified according to standard case definitions, linked into clusters epidemiologically, and reported to the Centers for Disease Control and Prevention.^10,11^ We analyzed available information on all confirmed cases of measles in the United States from January 1, 2001, to December 31, 2017. Data were collected as part of standardized public health surveillance and determined by the Centers for Disease Control and Prevention not to be research involving human participants.

In this cross-sectional study, we measured the transmissibility of measles by estimation of the effective reproduction number (*R*), or mean number of secondary cases of measles generated per single infectious individual in a population with some level of immunity (the basic reproduction number, *R*_0_, describes transmissibility in a fully susceptible population). Sustaining measles elimination requires maintenance of *R* below the threshold value of 1. If *R* is greater than 1, on average, each person spreads measles to more than 1 other person, and a self-sustaining outbreak can occur; by contrast, if *R* is less than 1, on average, each person spreads measles to less than 1 other person, and transmission cannot be sustained. Building on previous analyses,^12,13^ we adapted an existing algorithm^14,15^ that uses a maximum likelihood procedure to infer *R* for each case, or cohort of cases, given the time in days between cases in an outbreak and the probability density function of the serial interval (time between the onset of symptoms in primary cases of measles and the secondary cases they generate).^14,15^ We used a serial interval for measles derived from household transmission studies with a γ probability distribution and a mean (SD) of 11.1 (2.5) days.^16^ In brief, in any given measles case series, the weight that patient *i* infected patient *j*, *W_ij_*, is the serial interval distribution applied to the number of days between the rash onsets of patients *i* and *j*, and the probability that patient *j* was infected by patient *i*, *P_ij_*, is given by *P_ij_* = *W_ij_*/(∑*_k_W_kj_*), where the sum in the denominator is over all potential infectors *k* of patient *j*. The estimate of the *R* for patient *i* is *R_i_* = ∑*_j_P_ij_* (eMethods in the Supplement).

We applied the method to measles surveillance data by performing the procedure for all cases of measles after the index case (first identified case in a transmission chain) in each reported cluster of cases (2-case chains and outbreaks of ≥3 cases). The algorithm assigns singleton cases (single cases with no other cases epidemiologically linked to them) an *R* of 0.

Chains of transmission in which 2 consecutive cases of measles are too close or too far away in time based on the distribution of the serial interval and that are unexplained by other cases in the outbreak are likely to be an artifact of surveillance (eg, an unidentified common source or a missing case in a chain) and may erroneously be considered a transmission pair by the model. To account for this possibility, if a secondary case could not be ascribed to a case of measles presenting 6 through 18 days prior (ie, the observed range of serial interval values^17^ and equivalent to the central 95% CI profile of the serial interval),^16^ the supposed connection was excluded and the secondary case was reassigned as an index case and the procedure continued. This method allowed for inclusion in the analyses of all transmissions before and after the supposed transmission between consecutive cases.

When there is more than 1 measles case in any particular day of an outbreak, the method averages the number of forward transmissions originating from the cohort of patients with measles presenting that day,^14^ thereby increasing or decreasing the estimated contribution of any given case to transmission. However, the resolution of cases driving the transmission in each cohort can be improved by weighting the transmissibility of each case in a given day by characteristics associated to measles transmissibility. We weighted the transmissibility of each case in a given day by the number of doses of a measles-containing vaccine received (0, 1, ≥2, or unknown) and whether the patient was born before 1957 or on or after 1957. We adjusted transmissibility specifically by these 2 factors because receipt of measles vaccine and birth in the prevaccine era (ie, before 1957) are considered presumptive evidence of measles immunity,^11^ and levels of immunity are thought to be linked to the capacity to transmit the virus (eMethods and eTable 1 in the Supplement).^9,18,19^

We assessed *R* based on the following characteristics of patients with measles: vaccination status (0, 1, or ≥2 doses or unknown), birth prior to 1957 (presumed immune from natural exposure), sex, importation status (imported or US-acquired), residency status (US resident or foreign visitor), age (in months) at first dose, time (years) since vaccination, hospitalization, presence of complications, reporting US state, and genotype. We dichotomized age at first dose (<15 or ≥15 months), as previous studies indicate reduced antibody responses and increased susceptibility to measles when the first dose is given before 15 months of age.^6,7^ To evaluate for changes in transmissibility due to waning immunity, we dichotomized time since vaccination (<12 or ≥12 years), with 12 years being the median number of years since vaccination for available data. In addition, we evaluated *R* based on the vaccination status (0 doses, ≥1 dose, or unknown) and age group (<1, 1-4, 5-17, 18-29, 30-49, and ≥50 years) of both primary cases of measles and the secondary cases of measles that they generated.

We describe the demographic and epidemiologic characteristics of potential superspreading events, defined as a case with an estimated *R* greater than or equal to 5 (≥99th percentile of all estimates in this data set). Sensitivity analyses were performed to examine the choice of the measles serial interval, the width of the time window for allowable connections to be made between consecutive cases, and the characteristics included in the weighting procedure to determine the factors associated with transmission (eResults in the Supplement).

## Results

From 2001 to 2017, a total of 2218 confirmed measles cases were reported in the United States. Of these, 490 were single cases, 90 were 2 case-chains, and 116 were outbreaks of 3 or more cases. The median size of outbreaks was 5 cases (range, 3-383 cases) and median duration of outbreaks was 22 days (range, 3-121 days). Among the 2218 measles cases, 573 (25.8%) were internationally imported and 1645 (74.2%) were acquired in the United States. Most patients with measles were unvaccinated (1508 [68.0%]) or had an unknown vaccination status (435 [19.6%]). The date of vaccine receipt was poorly populated in our data set (available for 100 of 275 vaccinated individuals [36.4%]). Additional key characteristics of measles cases are shown in eTable 2 in the Supplement. A graphical representation of the transmission matrix for one outbreak is shown in the eFigure in the Supplement.

**Figure 1.**
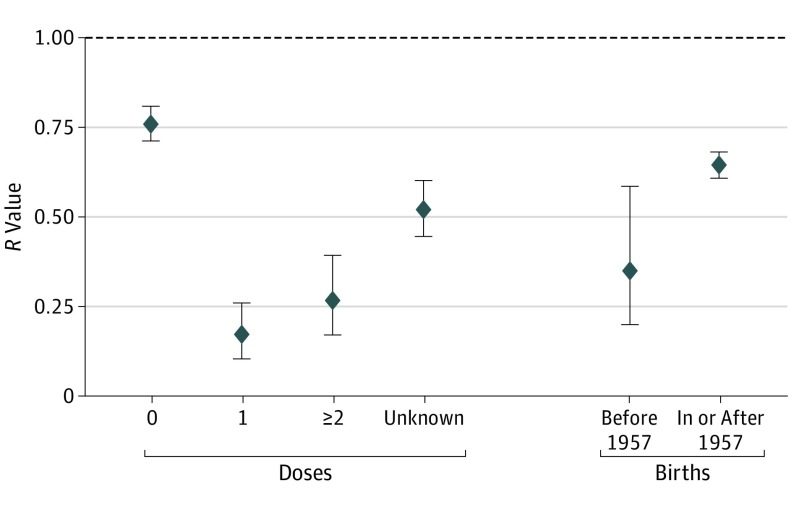
Estimates of the Measles Case Reproduction Number, *R*, by Vaccination Status and Birth Before 1957 The bars represent the 95% CIs, and the horizontal dashed line indicates the threshold value of *R* = 1. Results are self-consistently adjusted by the number of doses of a measles-containing vaccine received and birth before 1957.

Estimates of *R* for measles in the United States were 0.76 (95% CI, 0.71-0.81) among patients who had received no doses of a measles-containing vaccine, 0.17 (95% CI, 0.11-0.26) among patients who had received 1 dose, 0.27 (95% CI, 0.17-0.39) among patients who had received 2 or more doses, and 0.52 (95% CI, 0.44-0.60) among those who had an unknown vaccination status. Among patients born before 1957, *R* was 0.35 (95% CI, 0.20-0.58), and among those born on or after 1957, *R* was 0.64 (95% CI, 0.61-0.68) (Figure 1).

Among unvaccinated primary cases of measles in patients who infected unvaccinated and vaccinated (≥1 doses) secondary cases of measles, *R* estimates were 0.61 (95% CI, 0.57-0.65) among unvaccinated individuals and 0.06 (95% CI, 0.05-0.08) among vaccinated individuals. Among vaccinated primary cases of measles in patients who infected unvaccinated and vaccinated secondary cases of measles, *R* estimates were 0.10 (95% CI, 0.06-0.15) among unvaccinated individuals and 0.07 (95% CI, 0.04-0.11) among vaccinated individuals (Table 1).

**Table 1.** Estimates of the Measles Reproduction Number, *R*, Among Primary and Secondary Cases of Measles, by Vaccination Status^a^

Vaccination Status of Primary Cases	Vaccination Status of Secondary Cases, *R* (95% CI)		
	Unknown Dose(s)^b^	0 Doses^b^	≥1 Dose(s)^b^
Unknown dose(s)^b^	0.16 (0.12-0.20)	0.27 (0.22-0.34)	0.09 (0.06-0.12)
0 Doses^b^	0.09 (0.08-0.11)	0.61 (0.57-0.65)	0.06 (0.05-0.08)
≥1 Dose(s)^b^	0.05 (0.03-0.09)	0.10 (0.06-0.15)	0.07 (0.04-0.11)

^a^ Results are self-consistently adjusted by the number of doses of a measles-containing vaccine received and birth before 1957.

^b^ Doses of a measles-containing vaccine; doses were counted if given at least 1 maximum incubation period (21 days) prior to the onset of rash.

Transmission was generally assortative by age groups (ie, transmission tended to be higher between individuals of a similar age group). *R* estimates were higher when primary and secondary cases of measles were patients aged 5 to 17 years (0.36 [95% CI, 0.31-0.42]) compared with assortative transmission in other age groups (<1 year, 0.14 [95% CI, 0.10-0.20]; 1-4 years, 0.25 [95% CI, 0.20-0.30]; 18-29 years, 0.19 [95% CI, 0.15-0.24]; 30-49 years, 0.15 [95% CI, 0.11-0.20]; ≥50 years, 0.04 [95% CI, 0.01-0.10]) (Table 2).

**Table 2.** Estimates of the Measles Reproduction Number, *R*, Among Primary and Secondary Cases of Measles, by Age Group^a^

Age Group of Primary Cases	Age Group of Secondary Cases, *R* (95% CI)					
	<1 y	1-4 y	5-17 y	18-29 y	30-49 y	≥50 y
<1 y	0.14 (0.10-0.20)	0.12 (0.08-0.19)	0.09 (0.05-0.15)	0.07 (0.04-0.12)	0.05 (0.03-0.10)	0.009 (0.002-0.03)
1-4 y	0.08 (0.05-0.12)	0.25 (0.20-0.30)	0.14 (0.11-0.19)	0.09 (0.06-0.12)	0.10 (0.07-0.14)	0.02 (0.008-0.04)
5-17 y	0.04 (0.03-0.07)	0.10 (0.08-0.14)	0.36 (0.31-0.42)	0.12 (0.09-0.16)	0.11 (0.08-0.14)	0.01 (0.005-0.03)
18-29 y	0.06 (0.04-0.09)	0.10 (0.07-0.13)	0.19 (0.15-0.25)	0.19 (0.15-0.24)	0.13 (0.09-0.17)	0.02 (0.01-0.05)
30-49 y	0.05 (0.03-0.08)	0.07 (0.04-0.10)	0.11 (0.08-0.16)	0.11 (0.08-0.16)	0.15 (0.11-0.20)	0.02 (0.01-0.04)
≥50 y	0.07 (0.03-0.15)	0.07 (0.03-0.16)	0.12 (0.06-0.24)	0.13 (0.06-0.25)	0.14 (0.07-0.25)	0.04 (0.01-0.10)

^a^ Results are self-consistently adjusted by the number of doses of a measles-containing vaccine received and birth before 1957.

**Figure 2.**
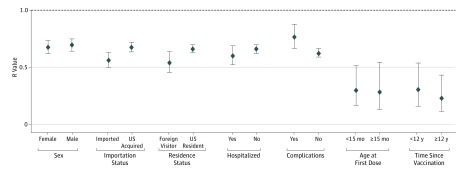
Estimates of the Measles Case Reproduction Number, *R*, by Various Case Characteristics The bars represent the 95% CIs, and the horizontal dashed line indicates the threshold value of *R* = 1. Results are self-consistently adjusted by the number of doses of a measles-containing vaccine received and birth before 1957.

**Figure 3.**
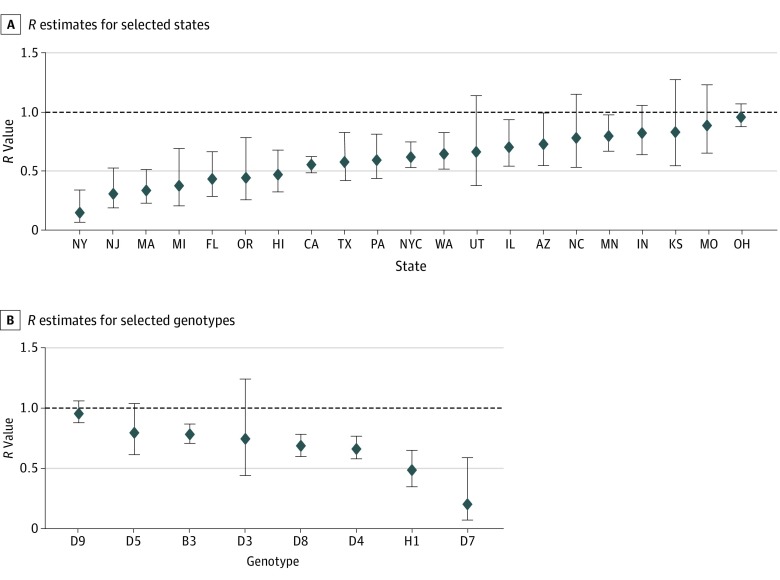
Estimates of the Measles Case Reproduction Number, *R*, by Reporting State and Genotype A, *R* estimates for selected states. Forty-seven US states, Washington, DC, and New York City reported measles cases during the study period. New York City Department of Health and Mental Hygiene and New York State Department of Health report separately. State data shown are for localities reporting 20 or more cases of measles, ordered by *R* point estimates: Utah (UT), 22; Michigan (MI), 28; Oregon (OR), 28; Kansas (KS), 29; North Carolina (NC), 32; New York (NY), 41; New Jersey (NJ), 42; Florida (FL), 48; Missouri (MO), 48; Hawaii (HI), 58; Texas (TX), 59; Pennsylvania (PA), 63; Arizona (AZ), 64; Massachusetts (MA), 68; Illinois (IL), 70; Indiana (IN), 73; Washington (WA), 105; Minnesota (MN), 121; New York City (NYC), 213; Ohio (OH), 397; and California (CA), 419. Measles cases are reported by state of residence, which may not necessarily be where the infection was acquired. B, *R* estimates for selected genotypes. Genotype data shown are for 8 genotypes identified in 15 or more cases, ordered by *R* point estimates: D9, 439; D5, 68; B3, 530; D3, 19; D8, 339; D4, 264; H1, 86; and D7, 15. The bars represent the 95% CIs, and the horizontal dashed line indicates the threshold value of *R* = 1. Results are self-consistently adjusted by the number of doses of a measles-containing vaccine received and birth before 1957.

Estimates of *R* were not substantially different based on sex, residence status, hospitalization, age at first dose, or time since vaccination (Figure 2). Estimates of *R* among patients who acquired measles abroad was estimated to be 0.56 (95% CI, 0.50-0.62) and among patients who acquired measles in the United States to be 0.67 (95% CI, 0.63-0.71). Estimates of *R* among patients reporting complications was 0.76 (95% CI, 0.66-0.88) and among those not reporting complications was 0.62 (95% CI, 0.59-0.66). Some differences in *R* estimates were seen based on the genotype and reporting state (Figure 3); some of these estimates were based on few cases, and most 95% CIs overlapped.

We identified 23 possible superspreading events during the study period (median *R* = 6.1 [range, 5.0-18.1]) (eTable 3 in the Supplement). The median age of superspreaders was 17 years (range, 9 months-63 years). Nineteen of the 23 individuals (82.6%) were unvaccinated (the remaining 4 had an unknown vaccination status), and 22 (95.7%) were born on or after 1957. Superspreading events occurred during 16 outbreaks (typically early in the outbreak), which had a median size of 21 cases (range, 6-383 cases) and median duration of 44 days (range, 18-121 days). Superspreading events occurred mostly in close-contact settings (eg, hospitals, households, and schools), and most individuals with measles reported in these outbreaks were unvaccinated.

Sensitivity analyses showed that varying several of the assumptions in this evaluation resulted in only small changes in general patterns of transmission (eTables 4-12 in the Supplement).

## Discussion

By pooling means of *R* based on various case characteristics, we were able to discern the factors associated with measles transmission in this postelimination setting. Principally, we found a gradient of transmission in which unvaccinated patients with measles are approximately 3 to 4 times more infectious compared with patients with measles who have been vaccinated once or twice, and that transmission is concentrated among unvaccinated primary and secondary cases of measles. Furthermore, our description of superspreading events highlight lack of vaccination as the initial spark for large outbreaks of measles. Although the measles vaccine is known to be highly effective in decreasing measles susceptibility—1 dose is 93% effective against measles and 2 doses are 97% effective—our findings also suggest an association of vaccination with limiting measles communicability and underscore the fact that measles transmission in the United States is driven by failure to vaccinate rather than a failure of vaccine performance. In addition, the low transmissibility observed from adults born when measles was still endemic (assumed to be naturally infected) supports the use of birth before vaccine introduction as acceptable presumptive evidence of measles immunity in elimination settings.^11^

Measles transmission was assortative with age (among persons aged <50 years, approximately 30%-50% of transmission events occurred within the same age group), consistent with age-specific mixing reported in studies that quantify social encounters that are potentially infectious.^3^ A key feature associated with the preferential interaction within age groups is the finding of more pronounced contacts among school-aged children (relative to contacts between adults).^3^ Our evaluation similarly shows school-aged children as a primary conduit of measles transmission in the United States and emphasizes the importance of policies aimed at ensuring high 2-dose vaccine coverage of these children (eg, school entry immunization requirements) or presumptive communication (informing parents that vaccines are scheduled during the visit) instead of participatory communication (asking parents if they would like their children to be vaccinated) during parent-clinician encounters.^20^ Age-specific *R* estimates derived from the probabilistic model^14,15^ could help clarify the extent by which social contact patterns explain disease transmission.^21^

*R* estimates among vaccinated patients with measles were generally very low, including toward unvaccinated cases (*R* = 0.10). These estimates might be biased because we did not differentiate between primary vaccine failure (failure to seroconvert after vaccination) and secondary vaccine failure (waning of immunity after seroconversion), and cases of measles owing to primary vaccine failure might be as transmissible as cases of measles in unvaccinated individuals. Individuals with secondary vaccine failure have a vigorous amnestic response to measles and thus might have milder symptoms and shed less virus.^9,18,22^ The presence of complications (a marker of disease severity) was independently associated with measles contagiousness. The largely restricted transmission of measles from vaccinated persons is in agreement with previous observations of no transmission from twice-vaccinated individuals with measles who develop robust antibody responses (despite exposing numerous persons).^9,18,19^ Because almost all persons who do not respond to the first dose of measles vaccine are expected to develop protective immunity after the second dose, our study provides further evidence for use of a 2-dose schedule in elimination efforts. Because measles antibody titers are known to decline slowly after measles vaccination,^8^ continued monitoring of measles among vaccinated persons is warranted in low-incidence settings.

There were subtle differences in transmissibility based on other factors. Compared with measles among foreign visitors and imported cases of measles, *R* values for cases of measles among US residents and US-acquired cases of measles tended to be higher. This finding might reflect the transitory nature of stays by foreign visitors and that they are less likely to contact local at-risk communities. Although some differences were also noted in *R* point estimates based on genotype and reporting state, 95% CIs overlapped for many of these estimates. The results presented here do not indicate that genotype B3 has increased transmissibility compared with other genotypes.^23^ Because the chance of measles spreading is dependent on the setting in which measles is introduced, differences in the observed transmissibility of a given genotype should be interpreted cautiously. For example, the higher *R* for Ohio and D9 is associated with an outbreak in an Amish community in 2014,^24^ likely owing to this community being highly underimmunized rather than to any characteristic of the virus (excluding this outbreak, the *R* estimates for Ohio was 0.16 and for D9 was 0.71). Other genotypes have been associated with large outbreaks in other settings (eg, H1 in Mongolia^25^ and D4 in France),^26^ and importations of these genotypes might have led to a similar outbreak in other underimmunized populations and would not have changed public health response efforts. Estimation of *R* associated with specific outbreaks can nonetheless serve as a marker of the extent of a particular immunity gap,^27^ and careful characterization of these susceptible communities can help pinpoint areas in which preventive interventions might be needed.^28^

### Limitations

Our study has some limitations. The algorithm^14,15^ does not conclusively establish who infected whom and cannot replace careful epidemiologic investigation, but it is useful in identifying the overall direction of transmission. Because the likelihood of transmission depends on several factors, including the status (eg, vaccination) of both the infector and infectee, the setting in which the exposure occurred, and outbreak containment interventions, it is challenging to account for the effect of each potential confounder. For example, we did not directly evaluate the association of population density with transmission of measles, although our analysis of superspreading events indicates that close-contact settings provide opportunities for rapid dissemination of measles. Similarly, we did not evaluate the association of clustering with transmission of measles, and geographic clustering of unvaccinated persons has been linked to measles outbreaks.^24,29,30^ Unvaccinated primary cases of measles were more likely to infect unvaccinated rather than vaccinated individuals, whereas vaccinated primary cases of measles infected a similar number of unvaccinated and vaccinated individuals. Furthermore, the range of *R* values during superspreading events was similar to the commonly cited range of values for *R*_0_.^27^ Both observations imply that there are pockets of underimmunization in the United States. The date of vaccine receipt was poorly populated in our data set (available for approximately 36% of vaccinated cases), and our results of no difference in transmissibility by age at first dose and time since vaccination were based on few cases. These findings were also confounded by lack of differentiation between primary and secondary vaccine failure, which requires specialized testing (avidity and neutralizing antibody titers). However, our analyses suggest that vaccinated persons are inefficient transmitters of measles, and we found no notable differences in transmissibility between vaccinated individuals with measles with and without reported vaccination dates (eTable 13 in the Supplement). The outbreaks we evaluated occurred in diverse populations and were affected by several individual- and context-specific factors; thus, the relative importance of the different factors associated with transmission might not be generalizable. Finally, our comparisons of *R* values were qualitative and not statistical, although clear differences in transmissibility were noted and explained by underlying covariates.

## Conclusions

The method^14,15^ we used allowed us to identify leading factors associated with the spread of measles in an elimination setting from high-quality surveillance data. Our findings show predominantly subcritical (*R *< 1) transmission of measles and maintenance of elimination in the United States for the past 17 years,^12^ establish the public health value of the measles vaccine in limiting measles infectiousness, and underscore the importance of having high targets for 2-dose measles vaccine coverage, especially among school-aged children.

## Notes

Supplement. **eMethods.** Additional Details of the Data Used for Analyses **eResults.** Comparison of Unadjusted and Adjusted Estimates of *R* **eTable 1.** Results of a Multiple Linear Regression Model to Assess the Relationship Between *R* and Several Case Characteristics **eTable 2.** Key Characteristics of 2218 Measles Case-Patients Reported in the United States, 2001-2017 **eTable 3.** Summary Characteristics of 23 Potential Measles Superspreading Events During 16 Measles Outbreaks **eTable 4.** Estimates of the Measles Case Reproduction Number, *R*, According to Several Characteristics, Adjusting Transmissibility by Various Covariates, United States, 2001-2017 **eTable 5.** Estimates of the Measles Case Reproduction Number, *R*, Among Primary and Secondary Cases According to Vaccination Status, Adjusting Transmissibility by Various Covariates, United States, 2001-2017 **eTable 6.** Estimates of the Measles Case Reproduction Number, *R*, According to Age Group, Adjusting Transmissibility by Various Covariates, United States, 2001-2017 **eTable 7.** Estimates of the Measles Case Reproduction Number, *R*, According to Several Characteristics, Estimated Using Three Different Serial Intervals, United States, 2001-2017 **eTable 8.** Estimates of the Measles Case Reproduction Number, *R*, Among Primary and Secondary Cases, by Vaccination Status, Estimated Using Three Different Serial Intervals, United States, 2001-2017 **eTable 9.** Measles Case Reproduction Numbers, *R*, Among Primary and Secondary Cases, by Age Groups, Estimated Using Three Different Serial Intervals, United States, 2001-2017 **eTable 10.** Estimates of the Measles Case Reproduction Number, *R*, According to Several Characteristics, Estimated Using Different Minimum and Maximum Serial Intervals, United States, 2001-2017 **eTable 11.** Estimates of the Measles Case Reproduction Number, R, Among Primary and Secondary Cases, by Vaccination Status, Estimated Using Different Minimum and Maximum Serial Intervals, United States, 2001-2017 **eTable 12.** Measles Case Reproduction Numbers, *R*, Among Primary and Secondary Cases, by Age Groups, Estimated Using Different Minimum and Maximum Serial Intervals, United States, 2001-2017 **eTable 13.** Estimates of the Measles Case Reproduction Number, *R*, Among Vaccinated Cases With and Without Dates of Vaccination Reported, United States, 2001-2017 **eFigure.** Outbreak Transmission Matrix
