# Supplementary material for: Factors Associated With Measles Transmission in the United States During the Postelimination Era
Source: JAMA Pediatr. 2019 Nov 18;174(1):56–62. doi: 10.1001/jamapediatrics.2019.4357 (PMC6865326; doi:10.1001/jamapediatrics.2019.4357)
Supplement: Supplement. — eMethods. Additional Details of the Data Used for Analyses eResults. Comparison of Unadjusted and Adjusted Estimates of R eTable 1. Results of a Multiple Linear Regression Model to Assess the Relationship Between R and Several Case Characteristics eTable 2. Key Characteristics of 2218 Measles Case-Patients Reported in the United States, 2001-2017 eTable 3. Summary Characteristics of 23 Potential Measles Superspreading Events During 16 Measles Outbreaks eTable 4. Estimates of the Measles Case Reproduction Number, R, According to Several Characteristics, Adjusting Transmissibility by Various Covariates, United States, 2001-2017 eTable 5. Estimates of the Measles Case Reproduction Number, R, Among Primary and Secondary Cases According to Vaccination Status, Adjusting Transmissibility by Various Covariates, United States, 2001-2017 eTable 6. Estimates of the Measles Case Reproduction Number, R, According to Age Group, Adjusting Transmissibility by Various Covariates, United States, 2001-2017 eTable 7. Estimates of the Measles Case Reproduction Number, R, According to Several Characteristics, Estimated Using Three Different Serial Intervals, United States, 2001-2017 eTable 8. Estimates of the Measles Case Reproduction Number, R, Among Primary and Secondary Cases, by Vaccination Status, Estimated Using Three Different Serial Intervals, United States, 2001-2017 eTable 9. Measles Case Reproduction Numbers, R, Among Primary and Secondary Cases, by Age Groups, Estimated Using Three Different Serial Intervals, United States, 2001-2017 eTable 10. Estimates of the Measles Case Reproduction Number, R, According to Several Characteristics, Estimated Using Different Minimum and Maximum Serial Intervals, United States, 2001-2017 eTable 11. Estimates of the Measles Case Reproduction Number, R, Among Primary and Secondary Cases, by Vaccination Status, Estimated Using Different Minimum and Maximum Serial Intervals, United States, 2001-2017 eTable 12. Measles Case Reproduction Numbers, [file jamapediatr-174-56-s001.pdf]

Supplementary Online Content

Gastañaduy PA, Funk S, Lopman BA, et al. Factors associated with measles transmission in the United States during the postelimination era. *JAMA Pediatr.* Published online November 18, 2019. doi:10.1001/jamapediatrics.2019.4357

eMethods. Additional Details of the Data Used for Analyses
eResults. Comparison of Unadjusted and Adjusted Estimates of *R*
eTable 1. Results of a Multiple Linear Regression Model to Assess the Relationship Between *R* and Several Case Characteristics
eTable 2. Key Characteristics of 2218 Measles Case-Patients Reported in the United States, 2001-2017
eTable 3. Summary Characteristics of 23 Potential Measles Superspreading Events During 16 Measles Outbreaks
eTable 4. Estimates of the Measles Case Reproduction Number, *R*, According to Several Characteristics, Adjusting Transmissibility by Various Covariates, United States, 2001-2017
eTable 5. Estimates of the Measles Case Reproduction Number, *R*, Among Primary and Secondary Cases According to Vaccination Status, Adjusting Transmissibility by Various Covariates, United States, 2001-2017
eTable 6. Estimates of the Measles Case Reproduction Number, *R*, According to Age Group, Adjusting Transmissibility by Various Covariates, United States, 2001-2017
eTable 7. Estimates of the Measles Case Reproduction Number, *R*, According to Several Characteristics, Estimated Using Three Different Serial Intervals, United States, 2001-2017
eTable 8. Estimates of the Measles Case Reproduction Number, *R*, Among Primary and Secondary Cases, by Vaccination Status, Estimated Using Three Different Serial Intervals, United States, 2001-2017
eTable 9. Measles Case Reproduction Numbers, *R*, Among Primary and Secondary Cases, by Age Groups, Estimated Using Three Different Serial Intervals, United States, 2001-2017
eTable 10. Estimates of the Measles Case Reproduction Number, *R*, According to Several Characteristics, Estimated Using Different Minimum and Maximum Serial Intervals, United States, 2001-2017
eTable 11. Estimates of the Measles Case Reproduction Number, R, Among Primary and Secondary Cases, by Vaccination Status, Estimated Using Different Minimum and Maximum Serial Intervals, United States, 2001-2017
eTable 12. Measles Case Reproduction Numbers, *R*, Among Primary and Secondary Cases, by Age Groups, Estimated Using Different Minimum and Maximum Serial Intervals, United States, 2001-2017
eTable 13. Estimates of the Measles Case Reproduction Number, *R*, Among Vaccinated Cases With and Without Dates of Vaccination Reported, United States, 2001-2017
eFigure. Outbreak Transmission Matrix

This supplementary material has been provided by the authors to give readers additional information about their work.

© 2019 Gastañaduy PA et al. *JAMA Pediatrics*.

eMethods. Additional Details of the Data Used for Analyses

Confirmed cases of measles are either laboratory-confirmed or have a direct epidemiological linkage to a patient with laboratory-confirmed measles infection.[1,2] State health departments report confirmed cases of measles to the National Center for Immunization and Respiratory Diseases at the Centers for Disease Control and Prevention (CDC) via telephone or e-mail and electronically to the National Notifiable Diseases Surveillance System.[1,2]

Internationally imported cases are persons who were outside the U.S. during their exposure period (7-21 days before rash onset) and had rash onset within 21 days of entry into the U.S.; all other cases are considered U.S.-acquired.[1,2] The residence status of cases was categorized as either U.S.-resident or foreign visitor (e.g., international tourists and students, new international adoptees, recent immigrants).

Estimation procedure of the case reproduction number *R*

Estimation of the case reproduction number was done using the Wallinga and Teunis method.[3,4] We used a serial interval for measles derived from household transmission studies with a gamma probability distribution and a mean of 11·1 days and standard deviation of 2·47 days.[5] Consider a case series for an infectious disease where each person, except the index case, is infected by one other case. Let W_{ij} be the *a priori* weight that case j was infected by case i. W_{ij} may simply be a serial interval distribution applied to the number of days between the rash onset of cases i and j.

The probability, P_{ij}, that case j was infected by case i is

$$P_{ij} = \frac{W_{ij}}{\sum_k W_{kj}}, \tag{1}$$

where the sum in the denominator is over all potential infectors k of case j.

The expected value of the case reproduction number for case i is $R_i = \sum_j P_{ij}$. The expected value of the case reproduction number for a set, α, of n_α cases is $R_\alpha = \sum_{i\in\alpha} R_i/n_\alpha$.

Higher moments of the reproduction number for a set of cases may be easily estimated from a generating function, as follows. The probability that case j was infected by one of the cases in α is $P_{\alpha j} = \sum_{i\in\alpha} P_{ij}$. Define the generating function

$$G_{\alpha\beta}(x) = \prod_{j\in\beta} \left[\left(1 - P_{\alpha j}\right) + P_{\alpha j}x\right]. \tag{2}$$

The coefficient of x^n in $G_{\alpha\beta}(x)$ is the probability that the cases in α infected exactly n cases in the set β. (The sets α and β need not be mutually exclusive.) Numerical evaluation of the polynomial coefficients in $G_{\alpha\beta}(x)$ is straightforward. Let $G_{\alpha\beta}(x) = \sum_n p_n x^n$. Then,

$$R_{\alpha\beta} = \frac{1}{n_\alpha}\sum_n np_n \tag{3}$$

and

$$\mathrm{var}\left(R_{\alpha\beta}\right) = \frac{1}{n_\alpha}\sum_n n^2 p_n - \left(R_{\alpha\beta}\right)^2. \tag{4}$$

The cumulative probability distribution is $\pi_n = \sum_{m\leq n} p_m$. The q-th quantile of the reproduction number is the smallest value of n where $\pi_n \geq q$.

A likelihood based estimation of the reproduction number and its credible bounds proceeds as follows. If the number of transmissions due to a case is assumed to follow a Poisson distribution with mean r, and c cases are observed to result in n transmissions, then the normalized likelihood of r follows $\Gamma(n+1, c)$, a gamma distribution with shape $n+1$ and rate c. It follows that $R_{\alpha\beta}$ follows the distribution $\sum_n p_n \Gamma(n+1, c)/c$. Credible intervals of $R_{\alpha\beta}$ are given by appropriate quantiles of the distribution.

© 2019 Gastañaduy PA et al. *JAMA Pediatrics*.

Multivariable regression model to assess characteristics associated to *R*

Because several characteristics potentially associated to measles communicability are highly correlated, e.g., vaccination is related to both birth year and age, and disease severity is associated to both age and vaccination (complication rates are higher in persons aged <5 and >20 years[6] and disease is milder in vaccinated persons),[7,8] multivariable linear regression models were fit to examine case characteristics independently associated to *R*. Explanatory variables in the model included vaccination status, birth prior to 1957, age-groups, sex, importation status, residency status, hospitalization, and presence of complications. The following variables were independently associated to R_c when controlling for other covariates: vaccination status, birth before 1957, age-group, and complications (eTable1).

Although we identified four explanatory variables to be independently associated to measles transmission, incorporating them into the weighting procedure required some consideration. Some of these characteristics are expected to be related more to contact patterns than to an intrinsic capacity to transmit the virus, e.g., age-specific transmission might be more influenced by social contacts, while being vaccinated might also affect communicability by conferring some level of protection against symptoms. Because the procedure weights transmissibility by the characteristic of the infector and does not account for levels of susceptibility among contacts, the adjustment might not be applicable to factors that impact transmission as a result of a person's interactions. For this reason, due to concerns for collinearity, and because initial unadjusted results indicated differences in transmissibility were more marked based on vaccination and birth pre-vaccination, we chose to adjust our base analyses by these two factors. However, sensitivity analyses performed including more of these characteristics in the weighting procedure showed that patterns of transmissibility in more fully adjusted models were similar to those seen with our base analyses (eTables 4-6).

Adjustment of transmissibility based on characteristics of cases

The weights of cases with certain transmissibility characteristics α are assumed to be of the form $W_{ij} = \varphi_\alpha w_{ij}$ where φ_α is a transmissivity coefficient determined by the characteristics α of the primary cases (e.g., vaccination status, etc.), and are unknown. A self-consistent method to estimate the relative values of these coefficients is as follows. Assuming that the expected reproduction number for a case or a set of cases is proportional to the transmissivity coefficient for the case or set of cases, the relative values of the transmissibility coefficients may be estimated by solving

$$\frac{1}{n_\alpha}\sum_{i\in\alpha,j}\frac{\varphi_\alpha w_{ij}}{\sum_k \varphi_k w_{kj}} \propto \varphi_\alpha. \tag{5}$$

where n_α is number of cases with a given characteristic or set of characteristics α, φ_α is their transmissivity coefficient, and the sum is over all cases i with these characteristics, and all cases j that they could have infected.

Since only the relative values of the φ_i matter, one of them, say φ_z may be arbitrarily set to 1. Initiate all the φ to 1; in the next iteration, estimate

$$\varphi_\alpha \approx \frac{\frac{1}{n_\alpha}\sum_{i\in\alpha,j}\frac{\varphi_\alpha w_{ij}}{\sum_k \varphi_k w_{kj}}}{\frac{1}{n_z}\sum_{i\in z,j}\frac{w_{ij}}{\sum_k \varphi_k w_{kj}}}, \tag{6}$$

replace the estimated φ on the right hand side and iterate until the φ estimates converge.

© 2019 Gastañaduy PA et al. *JAMA Pediatrics*.

eResults. Comparison of Unadjusted and Adjusted Estimates of *R*

To investigate the effect of the weighting procedure that adjusts the relative transmissibility of cases presenting in a single day based on certain characteristics of these cases, we compared unadjusted estimates, to adjusted estimates including an increasing number of covariates in the weighting procedure. Results are shown in eTable 4-6.

Choice of the measles serial interval

Estimates of the measles case reproduction number, *R*, were derived from an existing algorithm[3,4] that uses the case incidence time series data (epidemic curves) and the distribution of the serial interval (the time between the onset of symptoms in primary cases and the secondary cases they generate). Our base analyses used a serial interval for measles derived from household transmission studies with a gamma probability distribution and a mean of 11·1 days and standard deviation of 2·47 days.[5] We also ran the analyses using two other (lower and higher) serial interval estimates reported in the literature.[9] Results are shown in eTables 7-9; base analyses results are shown for comparison.

Width of the time window for allowable connections to be made between consecutive cases.

When two consecutive cases in a chain of transmission are reported to be too near or distant from each other in regards to the number of days expected by the known distribution of the serial interval, this could be the result of either an unidentified common ancestor to these two cases, or an unidentified case between these two cases. In these scenarios, the algorithm might assign these cases as a transmission pair even though it might have been an issue of underreporting. To account for this, we excluded putative transmissions outside the observed range of reported serial intervals (approximately from 6 through 18 days),[9] which was also equivalent to the central 95% confidence interval (CI) profile of the serial interval we used. Here we examine results including all putative connections (no restriction analyses). Results are shown in eTables 10-12; the base analyses results are shown for comparison.

© 2019 Gastañaduy PA et al. *JAMA Pediatrics*.

eTable 1. Results of a Multiple Linear Regression Model to Assess the Relationship Between *R* and Several Case Characteristics

Case characteristic	Coefficient	95% CI	*P* value
**Vaccination status**			
0 doses	Referent		<0·04
1 dose	-0·40	(-0·59, -0·21)	
2 or more doses	-0·39	(-0·60, -0·18)	
Unknown	-0·15	(-0·29, -0·01)	
**Birth before 1957**			
No	Referent		0·05
Yes	-0·39	(-0·79 -0·0003)	
**Age-group**			
<1 year	Referent		<0·02
1-4 years	0·25	(0·07, 0·42)	
5-17 years	0·31	(0·14, 0·48)	
18-29 years	0·35	(0·17, 0·53)	
30-49 years	0·25	(0·06, 0·44)	
≥50 years	0·41	(0·09, 0·73)	
**Sex**			
Female	Referent		0·75
Male	0·02	(-0·08, 0·11)	
**Importation status**			
Imported	Referent		0·58
U.S.-acquired	0·04	(-0·09, 0·16)	
**Residence status**			
U.S.-resident	Referent		0·26
Foreign visitor	-0·10	(-0·26, 0·07)	
**Hospitalized**			
No	Referent		0·5
Yes	-0·04	(-0·18, 0·09)	
**Complications**			
No	Referent		0·010
Yes	0·18	(0·04, 0·32)	

95% CI=95% confidence intervals

© 2019 Gastañaduy PA et al. *JAMA Pediatrics*.

eTable 2. Key Characteristics of 2218 Measles Case-Patients Reported in the United States, 2001-2017[a]

Variable	Value
Male sex	1176/2201 (53)
Age years, median (range)[b]	15 (0, 89)
Age groups	
<1 year	247/2215 (11)
1-4 years	429/2215 (19)
5-17 years	548/2215 (25)
18-29 years	450/2215 (20)
30-49 years	443/2215 (20)
≥50 years	98/2215 (4)
Birth before 1957[c]	48/2216 (2)
Importation status	
Imported[d]	573/2218 (26)
U.S.-acquired[e]	1645/2218 (74)
Residence status[f]	
Foreign visitor	256/2214 (12)
U.S.-resident	1958/2214 (88)
Vaccination status	
0 doses[g]	1508/2218 (68)
1 dose[g]	155/2218 (7)
2 or more doses[g,h]	120/2218 (5)
Unknown	435/2218 (20)
Age at first dose[i]	
<15 months	51/91 (56)
≥15 months	40/91 (44)
Time since vaccination[j]	
<12 years	48/100 (48)
≥12 years	52/100 (52)
Complications[k]	328/2218 (15)
Hospitalization	426/2218 (19)
Genotypes[l]	
B3	530/1771 (30)
D9	439/1771 (25)
D8	339/1771 (19)
D4	264/1771 (15)
H1	86/1771 (5)
D5	68/1771 (4)
D3	19/1771 (1)
D7	15/1771 (1)
G3	7/1771 (0.4)
D6	3/1771 (0.2)
H2	1/1771 (0.1)

[a]The number of total measles case-patients differs among the characteristics because of varying completeness in reporting. Data were missing for the following variables: sex (17 cases), age in years (3 cases), age categories (3 cases), year of birth (2 cases), residence status (4 cases), and genotype (311 singleton cases, 32 two-case chains, and 18 outbreaks of 3 or more cases); information on the age at first dose and on the time since vaccination was available for 101 (37%) and 100 (36%) of 275 vaccinated case-patients, respectively.

[b]The median age is based on 2215 measles case-patients.

[c]Measles vaccine was first licensed in the United States in 1963; persons born before 1957 are likely to have been infected naturally and are thus considered to have acceptable presumptive evidence of measles immunity in the United States.

[d]Internationally imported cases are persons who acquired measles outside of the United States and brought their infection into the United States; i.e., they were outside the United States during their exposure period (7-21 days before rash onset), had rash onset within 21 days of entry into the United States, and had no known exposure to measles in the United States during that time.

© 2019 Gastañaduy PA et al. *JAMA Pediatrics*.

[e]U.S.-acquired are persons who had not been outside the United States during the 21 days before rash onset or who were known to have been exposed to measles within the United States.
[f]Categorized as either U.S.-resident or foreign visitor (e.g., international tourists and students, new international adoptees, recent immigrants).
[g]Of a measles-containing vaccine; doses were counted if given at least one maximum incubation period (21 days) prior to the onset of rash.
[h]Ten measles case-patients were reported to have received three doses, two to have received four doses, and one to have received five doses of a measles-containing vaccine.
[i]Previous studies indicate reduced antibody responses and increased susceptibility to measles when the first dose is given before 15 months of age (as opposed to when given at 15 months of age or older); the age at first dose was 6-8 months for 8 cases, 9-11 months for 6 cases, 12-14 months for 37 cases, 15-23 months for 21 cases, and 24-71 months for 19 cases.
[j]Time since last documented dose of a measles-containing vaccine; twelve years was the median value of time since vaccination.
[k]E.g., otitis media, diarrhea, vomiting, dehydration, pneumonia, thrombocytopenia, encephalitis, and death.
[l]Cases in a single chain of measles transmission that were not genotyped were assigned the same genotype as other cases in the chain.

© 2019 Gastañaduy PA et al. *JAMA Pediatrics*.

eTable 3. Summary Characteristics of 23 Potential Measles Superspreading Events During 16 Measles Outbreaks

**Variable**	**Value**
R, median (range)	6.1 (5.0, 18.1)
Age in years, median (range)	17 (0[a], 63)
Vaccination status	
0 doses	19/23 (83)
≥1 dose(s)	0/23 (0)
Unknown	4/23 (17)
Birth on or after 1957	22/23 (96)
Outbreak size, no. of cases, median (range)	21 (6-383)
Outbreak duration,[b] no. of days, median (range)	44 (18-121)
Outbreak day when event occurred, median (range)	1 (1, 33)[c]
Primary setting where outbreak occurred[d]	Households (10), school (4), healthcare (3)
Outbreaks with ≥80% of reported cases unvaccinated or with and unknown vaccination status	14/16 (88)[e]

R=measles case reproduction number.

[a]This measles case-patient was 9 months old.

[b]The duration of a chain of transmission was calculated as the difference between the dates of rash onset of the first and last cases.

[c]Seventeen or 74% of the 23 superspreading events occurred either in the first or second day of the outbreak.

[d]Other reported primary settings: Workplace (1), airplane (1), childcare (2), church (3), community (4); these settings are not mutually exclusive.

[e]Eleven or 69% of the 16 outbreaks had ≥80% of reported cases unvaccinated.

© 2019 Gastañaduy PA et al. *JAMA Pediatrics*.

eTable 4. Estimates of the Measles Case Reproduction Number, *R*, According to Several Characteristics, Adjusting Transmissibility by Various Covariates, United States, 2001-2017

Characteristic	*R* (95% CI)				
		Adjusted (variables included)			
	Unadjusted	Vaccination status	Vaccination status, birth before 1957 (base)	Vaccination status, birth before 1957, age-groups	Vaccination status, birth before 1957, age-groups, complications
**Vaccination status**					
0 doses	0·71 (0·67, 0·76)	0·75 (0·71, 0·80)	0·76 (0·71, 0·81)	0·74 (0·70, 0·79)	0·74 (0·69, 0·78)
1 dose	0·34 (0·24, 0·46)	0·17 (0·11, 0·26)	0·17 (0·11, 0·26)	0·19 (0·12, 0·28)	0·21 (0·14, 0·31)
2 or more doses	0·39 (0·27, 0·55)	0·27 (0·18, 0·4)	0·27 (0·17, 0·39)	0·29 (0·19, 0·42)	0·33 (0·23, 0·47)
Unknown	0·57 (0·50, 0·66)	0·53 (0·45, 0·61)	0·52 (0·44, 0·60)	0·56 (0·48, 0·64)	0·55 (0·48, 0·64)
**Birth before 1957**					
No	0·65 (0·62, 0·68)	0·65 (0·62, 0·68)	0·64 (0·61, 0·68)	0·65 (0·61, 0·68)	0·64 (0·61, 0·68)
Yes	0·38 (0·22, 0·62)	0·36 (0·21, 0·60)	0·35 (0·20, 0·58)	0·36 (0·21, 0·59)	0·36 (0·21, 0·59)
**Sex**					
Male	0·65 (0·61, 0·71)	0·65 (0·60, 0·70)	0·66 (0·61, 0·71)	0·66 (0·61, 0·71)	0·67 (0·62, 0·72)
Female	0·64 (0·59, 0·70)	0·64 (0·59, 0·70)	0·64 (0·58, 0·69)	0·63 (0·58, 0·69)	0·62 (0·57, 0·68)
**Importation status**					
Imported	0·56 (0·50, 0·63)	0·56 (0·50, 0·62)	0·56 (0·50, 0·62)	0·56 (0·50, 0·63)	0·56 (0·50, 0·63)
U.S.-acquired	0·67 (0·63, 0·71)	0·67 (0·63, 0·71)	0·67 (0·63, 0·71)	0·67 (0·63, 0·71)	0·67 (0·63, 0·71)
**Residence status**					
Foreign visitor	0·52 (0·44, 0·62)	0·54 (0·45, 0·64)	0·54 (0·45, 0·64)	0·54 (0·45, 0·64)	0·53 (0·44, 0·63)
U.S.-resident	0·66 (0·62, 0·70)	0·66 (0·62, 0·69)	0·66 (0·62, 0·69)	0·66 (0·62, 0·69)	0·66 (0·62, 0·70)
**Hospitalized**					
Yes	0·60 (0·53, 0·69)	0·60 (0·52, 0·69)	0·60 (0·52, 0·69)	0·61 (0·54, 0·70)	0·66 (0·58, 0·76)
No	0·65 (0·61, 0·69)	0·65 (0·62, 0·69)	0·65 (0·62, 0·69)	0·65 (0·61, 0·69)	0·64 (0·60, 0·68)

© 2019 Gastañaduy PA et al. *JAMA Pediatrics*.

**Complications**					
Yes	0·76 (0·66, 0·87)	0·76 (0·66, 0·88)	0·76 (0·66, 0·88)	0·78 (0·68, 0·89)	0·93 (0·82, 1·05)
No	0·62 (0·59, 0·66)	0·62 (0·59, 0·66)	0·62 (0·59, 0·66)	0·62 (0·58, 0·66)	0·59 (0·56, 0·63)
**Age at first dose**					
<15 months	0·48 (0·29, 0·76)	0·30 (0·17, 0·52)	0·30 (0·16, 0·51)	0·32 (0·18, 0·55)	0·34 (0·19, 0·57)
≥15 months	0·55 (0·31, 0·91)	0·28 (0·13, 0·55)	0·28 (0·12, 0·54)	0·28 (0·12, 0·54)	0·32 (0·16, 0·60)
**Time since vaccination**					
<12 years	0·56 (0·34, 0·87)	0·30 (0·16, 0·53)	0·30 (0·16, 0·53)	0·34 (0·19, 0·59)	0·38 (0·21, 0·64)
≥12 years	0·40 (0·22, 0·66)	0·23 (0·11, 0·44)	0·23 (0·11, 0·43)	0·21 (0·10, 0·40)	0·22 (0·11, 0·42)

© 2019 Gastañaduy PA et al. *JAMA Pediatrics*.

eTable 5. Estimates of the Measles Case Reproduction Number, *R*, Among Primary and Secondary Cases According to Vaccination Status, Adjusting Transmissibility by Various Covariates, United States, 2001-2017

			***R* (95% CI)**		
			**Doses(s)[a], secondary cases**		
			Unknown	0	1 or more
**Unadjusted**	**Dose(s)[a], primary cases**	Unknown	0·16 (0·13, 0·21)	0·32 (0·26, 0·39)	0·09 (0·06, 0·13)
		0	0·08 (0·07, 0·10)	0·58 (0·54, 0·62)	0·06 (0·04, 0·07)
		1 or more	0·09 (0·05, 0·14)	0·18 (0·12, 0·25)	0·09 (0·06, 0·14)
**Adjusted (variables included)**					
**Vaccination status**	**Dose(s)[a], primary cases**	Unknown	0·16 (0·12, 0·21)	0·28 (0·22, 0·35)	0·09 (0·06, 0·12)
		0	0·09 (0·08, 0·11)	0·60 (0·56, 0·64)	0·06 (0·05, 0·08)
		1 or more	0·05 (0·03, 0·09)	0·10 (0·06, 0·15)	0·07 (0·04, 0·11)
**Vaccination status, birth before 1957 (base)**	**Dose(s)[a], primary cases**	Unknown	0·16 (0·12, 0·20)	0·27 (0·22, 0·34)	0·09 (0·06, 0·12)
		0	0·09 (0·08, 0·11)	0·61 (0·57, 0·65)	0·06 (0·05, 0·08)
		1 or more	0·05 (0·03, 0·09)	0·10 (0·06, 0·15)	0·07 (0·04, 0·11)
**Vaccination status, birth before 1957, age-groups**	**Dose(s)[a], primary cases**	Unknown	0·17 (0·13, 0·21)	0·30 (0·24, 0·37)	0·09 (0·06, 0·13)
		0	0·09 (0·07, 0·11)	0·60 (0·56, 0·64)	0·06 (0·05, 0·07)
		1 or more	0·05 (0·03, 0·10)	0·10 (0·07, 0·16)	0·07 (0·05, 0·12)
**Vaccination status, birth before 1957, age-groups, complications**	**Dose(s)[a], primary cases**	Unknown	0·16 (0·12, 0·20)	0·31 (0·25, 0·38)	0·09 (0·06, 0·12)
		0	0·09 (0·07, 0·11)	0·59 (0·55, 0·63)	0·06 (0·05, 0·07)
		1 or more	0·07 (0·04, 0·11)	0·11 (0·07, 0·17)	0·08 (0·05, 0·13)

[a]Of a measles-containing vaccine; doses were counted if given at least one maximum incubation period (21 days) prior to the onset of rash.

© 2019 Gastañaduy PA et al. *JAMA Pediatrics*.

eTable 6. Estimates of the Measles Case Reproduction Number, *R*, According to Age Group, Adjusting Transmissibility by Various Covariates, United States, 2001-2017

	***R* (95% CI)**				
		**Adjusted (variables included)**			
**Age-groups (primary, secondary cases)**	**Unadjusted**	**Vaccination status**	**Vaccination status, birth before 1957 (base)**	**Vaccination status, birth before 1957, age-groups**	**Vaccination status, birth before 1957, age-groups, complications**
<1 year, <1 year	0·13 (0·09, 0·19)	0·14 (0·10, 0·20)	0·14 (0·10, 0·20)	0·13 (0·09, 0·18)	0·12 (0·09, 0·18)
<1 year, 1-4 years	0·12 (0·08, 0·18)	0·12 (0·08, 0·19)	0·12 (0·08, 0·19)	0·1 (0·06, 0·15)	0·09 (0·06, 0·15)
<1 year, 5-17 years	0·09 (0·05, 0·14)	0·09 (0·05, 0·15)	0·09 (0·05, 0·15)	0·06 (0·03, 0·10)	0·05 (0·03, 0·10)
<1 year, 18-29 years	0·07 (0·04, 0·12)	0·07 (0·04, 0·12)	0·07 (0·04, 0·12)	0·05 (0·03, 0·10)	0·05 (0·02, 0·09)
<1 year, 30-49 years	0·05 (0·02, 0·09)	0·05 (0·03, 0·10)	0·05 (0·03, 0·10)	0·04 (0·02, 0·08)	0·04 (0·02, 0·07)
<1 year, ≥50 years	0·009 (0·002, 0·03)	0·009 (0·002, 0·03)	0·009 (0·002, 0·03)	0·007 (0·001, 0·03)	0·007 (0·001, 0·03)
1-4 years, <1 year	0·08 (0·06, 0·12)	0·08 (0·05, 0·12)	0·08 (0·05, 0·12)	0·08 (0·05, 0·11)	0·08 (0·05, 0·11)
1-4 years, 1-4 years	0·24 (0·19, 0·30)	0·25 (0·20, 0·30)	0·25 (0·20, 0·30)	0·25 (0·20, 0·30)	0·25 (0·20, 0·30)
1-4 years, 5-17 years	0·15 (0·11, 0·19)	0·14 (0·11, 0·19)	0·14 (0·11, 0·19)	0·14 (0·10, 0·18)	0·14 (0·10, 0·19)
1-4 years, 18-29 years	0·08 (0·06, 0·12)	0·09 (0·06, 0·12)	0·09 (0·06, 0·12)	0·08 (0·05, 0·12)	0·08 (0·05, 0·12)
1-4 years, 30-49 years	0·1 (0·07, 0·14)	0·1 (0·07, 0·14)	0·1 (0·07, 0·14)	0·1 (0·07, 0·14)	0·11 (0·07, 0·15)
1-4 years, ≥50 years	0·02 (0·008, 0·04)	0·02 (0·008, 0·04)	0·02 (0·008, 0·04)	0·02 (0·007, 0·04)	0·02 (0·008, 0·04)
5-17 years, <1 year	0·04 (0·03, 0·07)	0·04 (0·03, 0·07)	0·04 (0·03, 0·07)	0·05 (0·03, 0·07)	0·05 (0·03, 0·07)
5-17 years, 1-4 years	0·1 (0·08, 0·14)	0·1 (0·08, 0·14)	0·1 (0·08, 0·14)	0·11 (0·08, 0·15)	0·11 (0·08, 0·14)
5-17 years, 5-17 years	0·36 (0·30, 0·42)	0·36 (0·31, 0·42)	0·36 (0·31, 0·42)	0·37 (0·31, 0·43)	0·37 (0·31, 0·43)
5-17 years, 18-29 years	0·12 (0·08, 0·15)	0·12 (0·09, 0·16)	0·12 (0·09, 0·16)	0·12 (0·09, 0·16)	0·12 (0·09, 0·16)
5-17 years, 30-49 years	0·1 (0·08, 0·14)	0·11 (0·08, 0·14)	0·11 (0·08, 0·14)	0·11 (0·08, 0·15)	0·11 (0·08, 0·14)
5-17 years, ≥50 years	0·01 (0·004, 0·03)	0·01 (0·005, 0·03)	0·01 (0·005, 0·03)	0·01 (0·005, 0·03)	0·01 (0·005, 0·03)
18-29 years, <1 year	0·06 (0·04, 0·09)	0·06 (0·04, 0·09)	0·06 (0·04, 0·09)	0·06 (0·04, 0·10)	0·07 (0·04, 0·10)
18-29 years, 1-4 years	0·1 (0·07, 0·14)	0·1 (0·07, 0·14)	0·1 (0·07, 0·13)	0·1 (0·07, 0·14)	0·1 (0·07, 0·14)

© 2019 Gastañaduy PA et al. *JAMA Pediatrics*.

	R (95% CI)					
		Adjusted (variables included)				
**Age-groups (primary, secondary cases)**	**Unadjusted**	**Vaccination status**	**Vaccination status, birth before 1957 (base)**	**Vaccination status, birth before 1957, age-groups**	**Vaccination status, birth before 1957, age-groups, complications**
18-29 years, 5-17 years	0·19 (0·15, 0·25)	0·19 (0·15, 0·25)	0·19 (0·15, 0·25)	0·21 (0·16, 0·26)	0·2 (0·16, 0·26)
18-29 years, 18-29 years	0·19 (0·15, 0·25)	0·19 (0·15, 0·24)	0·19 (0·15, 0·24)	0·20 (0·16, 0·25)	0·20 (0·15, 0·25)
18-29 years, 30-49 years	0·13 (0·09, 0·18)	0·13 (0·09, 0·17)	0·13 (0·09, 0·17)	0·14 (0·10, 0·18)	0·13 (0·09, 0·17)
18-29 years, ≥50 years	0·03 (0·01, 0·05)	0·02 (0·01, 0·05)	0·02 (0·01, 0·05)	0·03 (0·01, 0·05)	0·03 (0·01, 0·05)
30-49 years, <1 year	0·06 (0·04, 0·09)	0·05 (0·03, 0·08)	0·05 (0·03, 0·08)	0·05 (0·03, 0·08)	0·05 (0·03, 0·08)
30-49 years, 1-4 years	0·08 (0·05, 0·11)	0·07 (0·04, 0·11)	0·07 (0·04, 0·10)	0·07 (0·04, 0·10)	0·07 (0·04, 0·10)
30-49 years, 5-17 years	0·12 (0·09, 0·17)	0·12 (0·08, 0·16)	0·11 (0·08, 0·16)	0·11 (0·08, 0·15)	0·11 (0·07, 0·15)
30-49 years, 18-29 years	0·12 (0·08, 0·16)	0·11 (0·08, 0·16)	0·11 (0·08, 0·16)	0·11 (0·07, 0·15)	0·10 (0·07, 0·14)
30-49 years, 30-49 years	0·16 (0·12, 0·20)	0·15 (0·11, 0·20)	0·15 (0·11, 0·20)	0·14 (0·10, 0·18)	0·14 (0·10, 0·18)
30-49 years, ≥50 years	0·02 (0·01, 0·05)	0·02 (0·01, 0·05)	0·02 (0·01, 0·04)	0·02 (0·01, 0·04)	0·02 (0·01, 0·04)
≥50 years, <1 year	0·07 (0·03, 0·16)	0·07 (0·03, 0·16)	0·07 (0·03, 0·15)	0·08 (0·04, 0·17)	0·08 (0·04, 0·17)
≥50 years, 1-4 years	0·08 (0·03, 0·17)	0·07 (0·03, 0·16)	0·07 (0·03, 0·16)	0·09 (0·04, 0·19)	0·11 (0·05, 0·22)
≥50 years, 5-17 years	0·14 (0·07, 0·26)	0·12 (0·06, 0·24)	0·12 (0·05, 0·24)	0·15 (0·07, 0·28)	0·18 (0·10, 0·32)
≥50 years, 18-29 years	0·15 (0·08, 0·28)	0·14 (0·07, 0·24)	0·13 (0·06, 0·25)	0·18 (0·09, 0·31)	0·24 (0·14, 0·38)
≥50 years, 30-49 years	0·15 (0·08, 0·27)	0·14 (0·07, 0·25)	0·14 (0·07, 0·25)	0·17 (0·10, 0·30)	0·21 (0·12, 0·34)
≥50 years, ≥50 years	0·04 (0·01, 0·11)	0·04 (0·01, 0·11)	0·04 (0·01, 0·10)	0·05 (0·01, 0·12)	0·04 (0·01, 0·11)

© 2019 Gastañaduy PA et al. *JAMA Pediatrics*.

eTable 7. Estimates of the Measles Case Reproduction Number, *R*, According to Several Characteristics, Estimated Using Three Different Serial Intervals, United States, 2001-2017

Characteristic	*R* (95% CI)		
	Serial Interval Parameters		
	μ=11·1, σ=2·47 (base)	μ=9·9, σ=2·4	μ=13·8, σ=2·5
**Vaccination status**			
0 doses	0·76 (0·71, 0·81)	0·75 (0·71, 0·80)	0·74 (0·70, 0·79)
1 dose	0·17 (0·11, 0·26)	0·17 (0·10, 0·26)	0·14 (0·08, 0·22)
2 or more doses	0·27 (0·17, 0·39)	0·29 (0·19, 0·42)	0·25 (0·16, 0·37)
Unknown	0·52 (0·44, 0·60)	0·51 (0·43, 0·59)	0·51 (0·43, 0·59)
**Birth before 1957**			
No	0·64 (0·61, 0·68)	0·64 (0·61, 0·68)	0·63 (0·60, 0·66)
Yes	0·35 (0·20, 0·58)	0·34 (0·19, 0·57)	0·33 (0·19, 0·56)
**Sex**			
Male	0·66 (0·61, 0·71)	0·65 (0·60, 0·70)	0·65 (0·60, 0·70)
Female	0·64 (0·58, 0·69)	0·63 (0·58, 0·69)	0·61 (0·56, 0·67)
**Importation status**			
Imported	0·56 (0·50, 0·62)	0·51 (0·46, 0·58)	0·58 (0·52, 0·65)
U.S.-acquired	0·67 (0·63, 0·71)	0·68 (0·64, 0·72)	0·65 (0·61, 0·69)
**Residence status**			
Foreign visitor	0·54 (0·45, 0·64)	0·49 (0·41, 0·59)	0·55 (0·47, 0·65)
U.S.-resident	0·66 (0·62, 0·69)	0·66 (0·62, 0·69)	0·64 (0·60, 0·68)
**Hospitalized**			
Yes	0·60 (0·52, 0·69)	0·56 (0·49, 0·65)	0·62 (0·54, 0·71)
No	0·65 (0·62, 0·69)	0·66 (0·62, 0·70)	0·63 (0·59, 0·67)
**Complications**			
Yes	0·76 (0·66, 0·88)	0·75 (0·65, 0·86)	0·79 (0·69, 0·91)
No	0·62 (0·59, 0·66)	0·62 (0·58, 0·66)	0·60 (0·57, 0·64)
**Age at first dose**			
<15 months	0·30 (0·16, 0·51)	0·30 (0·16, 0·52)	0·26 (0·14, 0·46)
≥15 months	0·28 (0·12, 0·54)	0·31 (0·14, 0·58)	0·26 (0·12, 0·51)
**Time since vaccination**			
<12 years	0·30 (0·16, 0·53)	0·30 (0·15, 0·53)	0·26 (0·14, 0·48)
≥12 years	0·23 (0·11, 0·43)	0·25 (0·12, 0·46)	0·21 (0·10, 0·40)

μ, mean; σ, standard deviation.
Results are self-consistently adjusted by the number of doses of a measles-containing vaccine received and birth before 1957.

© 2019 Gastañaduy PA et al. *JAMA Pediatrics*.

eTable 8. Estimates of the Measles Case Reproduction Number, *R*, Among Primary and Secondary Cases, by Vaccination Status, Estimated Using Three Different Serial Intervals, United States, 2001-2017

			Doses(s)[a], secondary cases		
			Unknown	0	1 or more
**μ=11·1, σ=2·47 (base)**	Dose(s)[a], primary cases	Unknown	0·16 (0·12, 0·20)	0·27 (0·22, 0·34)	0·09 (0·06, 0·12)
		0	0·09 (0·08, 0·11)	0·61 (0·57, 0·65)	0·06 (0·05, 0·08)
		1 or more	0·05 (0·03, 0·09)	0·10 (0·06, 0·15)	0·07 (0·04, 0·11)
**μ=9·9, σ=2·4**	Dose(s)[a], primary cases	Unknown	0·16 (0·12, 0·20)	0·27 (0·21, 0·33)	0·08 (0·06, 0·12)
		0	0·09 (0·08, 0·11)	0·60 (0·56, 0·64)	0·06 (0·05, 0·07)
		1 or more	0·05 (0·03, 0·10)	0·10 (0·06, 0·15)	0·07 (0·04, 0·11)
**μ=13·8, σ=2·5**	Dose(s)[a], primary cases	Unknown	0·16 (0·12, 0·20)	0·26 (0·21, 0·33)	0·09 (0·06, 0·12)
		0	0·09 (0·08, 0·11)	0·59 (0·55, 0·63)	0·06 (0·05, 0·08)
		1 or more	0·04 (0·02, 0·08)	0·08 (0·05, 0·13)	0·06 (0·04, 0·10)

μ=mean; σ=standard deviation.
Results are self-consistently adjusted by the number of doses of a measles-containing vaccine received and birth before 1957.
[a]Of a measles-containing vaccine; doses were counted if given at least one maximum incubation period (21 days) prior to the onset of rash.

© 2019 Gastañaduy PA et al. *JAMA Pediatrics*.

eTable 9. Measles Case Reproduction Numbers, *R*, Among Primary and Secondary Cases, by Age Groups, Estimated Using Three Different Serial Intervals, United States, 2001-2017

			**Age-groups, secondary cases**					
			**<1 year**	**1-4 years**	**5-17 years**	**18-29 years**	**30-49 years**	**≥50 years**
**μ=11·1, σ=2·47 (base)**	Age-groups, primary cases	<1 year	0·14 (0·10, 0·20)	0·12 (0·08, 0·19)	0·09 (0·05, 0·15)	0·07 (0·04, 0·12)	0·05 (0·03, 0·10)	0·009 (0·002, 0·03)
		1-4 years	0·08 (0·05, 0·12)	0·25 (0·20, 0·30)	0·14 (0·11, 0·19)	0·09 (0·06, 0·12)	0·10 (0·07, 0·14)	0·02 (0·008, 0·04)
		5-17 years	0·04 (0·03, 0·07)	0·10 (0·08, 0·14)	0·36 (0·31, 0·42)	0·12 (0·09, 0·16)	0·11 (0·08, 0·14)	0·01 (0·005, 0·03)
		18-29 years	0·06 (0·04, 0·09)	0·10 (0·07, 0·13)	0·19 (0·15, 0·25)	0·19 (0·15, 0·24)	0·13 (0·09, 0·17)	0·02 (0·01, 0·05)
		30-49 years	0·05 (0·03, 0·08)	0·07 (0·04, 0·10)	0·11 (0·08, 0·16)	0·11 (0·08, 0·16)	0·15 (0·11, 0·20)	0·02 (0·01, 0·04)
		≥50 years	0·07 (0·03, 0·15)	0·07 (0·03, 0·16)	0·12 (0·06, 0·24)	0·13 (0·06, 0·25)	0·14 (0·07, 0·25)	0·04 (0·01, 0·10)
**μ=9·9, σ=2·4**	Age-groups, primary cases	<1 year	0·15 (0·10, 0·21)	0·12 (0·08, 0·18)	0·10 (0·06, 0·16)	0·07 (0·04, 0·12)	0·06 (0·03, 0·10)	0·01 (0·002, 0·04)
		1-4 years	0·08 (0·05, 0·11)	0·24 (0·20, 0·30)	0·14 (0·10, 0·19)	0·09 (0·06, 0·13)	0·10 (0·07, 0·14)	0·02 (0·006, 0·04)
		5-17 years	0·04 (0·03, 0·07)	0·10 (0·08, 0·14)	0·36 (0·31, 0·42)	0·12 (0·09, 0·16)	0·10 (0·08, 0·14)	0·01 (0·005, 0·03)
		18-29 years	0·06 (0·04, 0·09)	0·09 (0·06, 0·13)	0·20 (0·15, 0·25)	0·19 (0·14, 0·24)	0·13 (0·09, 0·17)	0·02 (0·01, 0·05)
		30-49 years	0·05 (0·03, 0·08)	0·07 (0·04, 0·11)	0·11 (0·08, 0·16)	0·11 (0·07, 0·15)	0·15 (0·11, 0·19)	0·02 (0·01, 0·04)
		≥50 years	0·08 (0·03, 0·17)	0·07 (0·03, 0·16)	0·13 (0·06, 0·24)	0·13 (0·06, 0·25)	0·12 (0·06, 0·23)	0·04 (0·01, 0·11)
**μ=13·8, σ=2·5**	Age-groups, primary cases	<1 year	0·14 (0·09, 0·19)	0·13 (0·08, 0·19)	0·09 (0·05, 0·15)	0·06 (0·04, 0·11)	0·05 (0·03, 0·09)	0·008 (0·002, 0·03)
		1-4 years	0·07 (0·05, 0·11)	0·25 (0·20, 0·31)	0·14 (0·10, 0·19)	0·08 (0·05, 0·12)	0·09 (0·07, 0·13)	0·02 (0·008, 0·04)
		5-17 years	0·04 (0·03, 0·07)	0·10 (0·07, 0·14)	0·33 (0·28, 0·39)	0·12 (0·09, 0·16)	0·10 (0·08, 0·14)	0·01 (0·004, 0·03)

© 2019 Gastañaduy PA et al. *JAMA Pediatrics*.

			Age-groups, secondary cases					
			**<1 year**	**1-4 years**	**5-17 years**	**18-29 years**	**30-49 years**	**≥50 years**
		18-29 years	0·06 (0·03, 0·09)	0·09 (0·07, 0·13)	0·19 (0·15, 0·24)	0·19 (0·15, 0·25)	0·13 (0·09, 0·17)	0·02 (0·01, 0·05)
		30-49 years	0·06 (0·04, 0·09)	0·06 (0·04, 0·10)	0·11 (0·08, 0·16)	0·11 (0·08, 0·15)	0·15 (0·11, 0·20)	0·02 (0·009, 0·04)
		≥50 years	0·07 (0·03, 0·15)	0·07 (0·02, 0·15)	0·11 (0·04, 0·22)	0·13 (0·06, 0·25)	0·16 (0·09, 0·27)	0·03 (0·01, 0·10)

μ, mean; σ, standard deviation.
Results are self-consistently adjusted by the number of doses of a measles-containing vaccine received and birth before 1957.

© 2019 Gastañaduy PA et al. *JAMA Pediatrics*.

eTable 10. Estimates of the Measles Case Reproduction Number, *R*, According to Several Characteristics, Estimated Using Different Minimum and Maximum Serial Intervals, United States, 2001-2017

Characteristics	*R* (95% CI)	
	Serial Interval Parameters (minimum, maximum)	
	6, 18 (base)	No Restriction
**Vaccination status**		
0 doses	0·76 (0·71, 0·81)	0·79 (0·74, 0·83)
1 dose	0·17 (0·11, 0·26)	0·29 (0·20, 0·40)
2 or more doses	0·27 (0·17, 0·39)	0·32 (0·22, 0·46)
Unknown	0·52 (0·44, 0·60)	0·56 (0·49, 0·65)
**Birth before 1957**		
No	0·64 (0·61, 0·68)	0·69 (0·65, 0·72)
Yes	0·35 (0·20, 0·58)	0·37 (0·22, 0·61)
**Sex**		
Male	0·66 (0·61, 0·71)	0·69 (0·64, 0·75)
Female	0·64 (0·58, 0·69)	0·68 (0·62, 0·73)
**Importation status**		
Imported	0·56 (0·50, 0·62)	0·58 (0·49, 0·68)
U.S.-acquired	0·67 (0·63, 0·71)	0·70 (0·66, 0·74)
**Residence status**		
Foreign visitor	0·54 (0·45, 0·64)	0·62 (0·56, 0·69)
U.S.-resident	0·66 (0·62, 0·69)	0·70 (0·66, 0·75)
**Hospitalized**		
Yes	0·60 (0·52, 0·69)	0·65 (0·57, 0·74)
No	0·65 (0·62, 0·69)	0·69 (0·65, 0·73)
**Complications**		
Yes	0·76 (0·66, 0·88)	0·83 (0·72, 0·94)
No	0·62 (0·59, 0·66)	0·66 (0·62, 0·70)
**Age at first dose**		
<15 months	0·30 (0·16, 0·51)	0·45 (0·28, 0·70)
≥15 months	0·28 (0·12, 0·54)	0·37 (0·19, 0·66)
**Time since vaccination**		
<12 years	0·30 (0·16, 0·53)	0·45 (0·27, 0·73)
≥12 years	0·23 (0·11, 0·43)	0·31 (0·17, 0·54)

Results are self-consistently adjusted by the number of doses of a measles-containing vaccine received and birth before 1957.

© 2019 Gastañaduy PA et al. *JAMA Pediatrics*.

eTable 11. Estimates of the Measles Case Reproduction Number, *R*, Among Primary and Secondary Cases, by Vaccination Status, Estimated Using Different Minimum and Maximum Serial Intervals, United States, 2001-2017

			Doses(s)[a], secondary cases		
			Unknown	0	1 or more
**No restriction**	Dose(s)[a], primary cases	Unknown	0·18 (0·14, 0·23)	0·29 (0·24, 0·36)	0·09 (0·06, 0·13)
		0	0·09 (0·08, 0·11)	0·63 (0·59, 0·67)	0·06 (0·05, 0·08)
		1 or more	0·06 (0·04, 0·11)	0·15 (0·11, 0·22)	0·08 (0·05, 0·13)
**6, 18 (base)**	Dose(s)[a], primary cases	Unknown	0·16 (0·12, 0·20)	0·27 (0·22, 0·34)	0·09 (0·06, 0·12)
		0	0·09 (0·08, 0·11)	0·61 (0·57, 0·65)	0·06 (0·05, 0·08)
		1 or more	0·05 (0·03, 0·09)	0·10 (0·06, 0·15)	0·07 (0·04, 0·11)

Results are self-consistently adjusted by the number of doses of a measles-containing vaccine received and birth before 1957.
[a]Of a measles-containing vaccine; doses were counted if given at least one maximum incubation period (21 days) prior to the onset of rash.

© 2019 Gastañaduy PA et al. *JAMA Pediatrics*.

eTable 12. Measles Case Reproduction Numbers, *R*, Among Primary and Secondary Cases, by Age Groups, Estimated Using Different Minimum and Maximum Serial Intervals, United States, 2001-2017

			Age-groups, secondary cases					
			<1 year	1-4 years	5-17 years	18-29 years	30-49 years	≥50 years
**No restriction**	Age-groups, primary cases	<1 year	0·14 (0·10, 0·20)	0·13 (0·08, 0·19)	0·09 (0·05, 0·15)	0·07 (0·04, 0·12)	0·05 (0·03, 0·10)	0·009 (0·002, 0·03)
		1-4 years	0·08 (0·06, 0·12)	0·28 (0·23, 0·34)	0·15 (0·11, 0·20)	0·10 (0·07, 0·14)	0·11 (0·08, 0·15)	0·02 (0·01, 0·04)
		5-17 years	0·05 (0·03, 0·07)	0·11 (0·08, 0·14)	0·37 (0·32, 0·43)	0·12 (0·09, 0·16)	0·11 (0·09, 0·15)	0·01 (0·006, 0·03)
		18-29 years	0·06 (0·04, 0·09)	0·10 (0·07, 0·13)	0·20 (0·16, 0·26)	0·21 (0·17, 0·26)	0·14 (0·10, 0·18)	0·03 (0·01, 0·05)
		30-49 years	0·06 (0·04, 0·09)	0·07 (0·05, 0·11)	0·12 (0·08, 0·16)	0·12 (0·09, 0·16)	0·16 (0·12, 0·21)	0·02 (0·01, 0·04)
		≥50 years	0·08 (0·03, 0·17)	0·07 (0·03, 0·17)	0·13 (0·06, 0·24)	0·16 (0·08, 0·28)	0·17 (0·10, 0·29)	0·05 (0·02, 0·12)
**6, 18 (base)**	Age-groups, primary cases	<1 year	0·14 (0·10, 0·20)	0·12 (0·08, 0·19)	0·09 (0·05, 0·15)	0·07 (0·04, 0·12)	0·05 (0·03, 0·10)	0·009 (0·002, 0·03)
		1-4 years	0·08 (0·05, 0·12)	0·25 (0·20, 0·30)	0·14 (0·11, 0·19)	0·09 (0·06, 0·12)	0·10 (0·07, 0·14)	0·02 (0·008, 0·04)
		5-17 years	0·04 (0·03, 0·07)	0·10 (0·08, 0·14)	0·36 (0·31, 0·42)	0·12 (0·09, 0·16)	0·11 (0·08, 0·14)	0·01 (0·005, 0·03)
		18-29 years	0·06 (0·04, 0·09)	0·10 (0·07, 0·13)	0·19 (0·15, 0·25)	0·19 (0·15, 0·24)	0·13 (0·09, 0·17)	0·02 (0·01, 0·05)
		30-49 years	0·05 (0·03, 0·08)	0·07 (0·04, 0·10)	0·11 (0·08, 0·16)	0·11 (0·08, 0·16)	0·15 (0·11, 0·20)	0·02 (0·01, 0·04)
		≥50 years	0·07 (0·03, 0·15)	0·07 (0·03, 0·16)	0·12 (0·06, 0·24)	0·13 (0·06, 0·25)	0·14 (0·07, 0·25)	0·04 (0·01, 0·10)

Results are self-consistently adjusted by the number of doses of a measles-containing vaccine received and birth before 1957.

© 2019 Gastañaduy PA et al. *JAMA Pediatrics*.

eTable 13. Estimates of the Measles Case Reproduction Number, *R*, Among Vaccinated Cases With and Without Dates of Vaccination Reported, United States, 2001-2017

**Vaccinated cases**[a]	***R* (95% CI)**	
	**Unadjusted**	**Adjusted**[b]
Without reported dates	0·30 (0·21, 0·41)	0·18 (0·12, 0·27)
With reported dates	0·47 (0·33, 0·67)	0·26 (0·16, 0·40)

[a]Of a measles-containing vaccine; doses were counted if given at least one maximum incubation period (21 days) prior to the onset of rash; includes 175 vaccinated cases without reported dates, and 100 vaccinated cases with reported dates.
[b]Results are self-consistently adjusted by the number of doses of a measles-containing vaccine received and birth before 1957.

© 2019 Gastañaduy PA et al. *JAMA Pediatrics*.

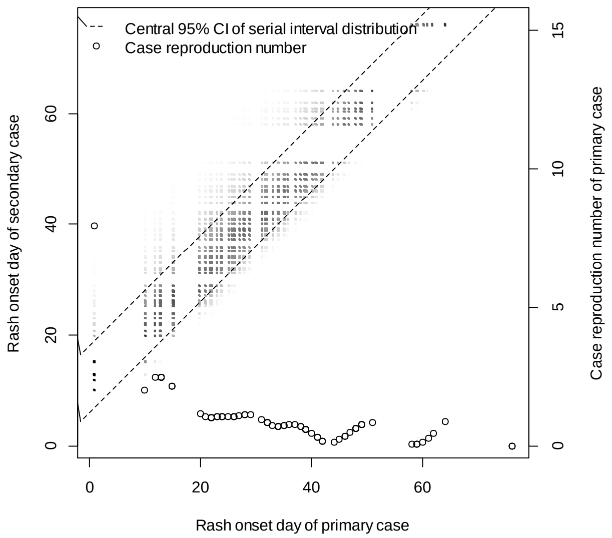

eFigure. Outbreak Transmission Matrix

A representation of the transmission matrix for a single outbreak showing the day of rash onset of primary cases in the horizontal axis, and the day of rash onset of secondary cases in the vertical axis. The squares represent all possible secondary cases arising from the primary case presenting that day; the probability of each primary case infecting (i.e., being the ancestor to) the secondary case are shown as squares in a gray-scale, with darker squares being the more likely ancestor. The dashed lines indicate the time lag corresponding to the central 95% confidence interval of the serial interval distribution, based on the rash onset of each primary case; thus darker squares (likely primary-secondary pairings) fall within these limits. The circles represent the reproduction number, *R*, assigned to each primary case, calculated as the sum of probabilities of it being the ancestor (over all secondary cases). As an example, the first case in the outbreak is assigned a *R*=7·9, equal to the sum of probabilities of the first case being the ancestor, over all secondary cases.

© 2019 Gastañaduy PA et al. *JAMA Pediatrics*.

eReferences

1. CDC. National Notifiable Disease Surveillance System (NNDSS). https://wwwn.cdc.gov/nndss/conditions/measles/case-definition/2013/ (accessed Feb 1 2019).
2. Gastanaduy P, Redd S, Clemmons N, et al. Manual for the Surveillance of Vaccine-Preventable Diseases. Chapter 7: Measles. April 6, 2018 2018. https://www.cdc.gov/vaccines/pubs/surv-manual/chpt07-measles.html (accessed May 6 2018).
3. Wallinga J, Teunis P. Different epidemic curves for severe acute respiratory syndrome reveal similar impacts of control measures. *Am J Epidemiol* 2004; **160**(6): 509-16.
4. Cori A, Ferguson NM, Fraser C, Cauchemez S. A new framework and software to estimate time-varying reproduction numbers during epidemics. *Am J Epidemiol* 2013; **178**(9): 1505-12.
5. Klinkenberg D, Nishiura H. The correlation between infectivity and incubation period of measles, estimated from households with two cases. *J Theor Biol* 2011; **284**(1): 52-60.
6. Perry RT, Halsey NA. The clinical significance of measles: a review. *J Infect Dis* 2004; **189 Suppl 1**: S4-16.
7. Hahne SJ, Nic Lochlainn LM, van Burgel ND, et al. Measles Outbreak Among Previously Immunized Healthcare Workers, the Netherlands, 2014. *J Infect Dis* 2016; **214**(12): 1980-6.
8. Rosen JB, Rota JS, Hickman CJ, et al. Outbreak of measles among persons with prior evidence of immunity, New York City, 2011. *Clin Infect Dis* 2014; **58**(9): 1205-10.
9. Vink MA, Bootsma MC, Wallinga J. Serial intervals of respiratory infectious diseases: a systematic review and analysis. *Am J Epidemiol* 2014; **180**(9): 865-75.

© 2019 Gastañaduy PA et al. *JAMA Pediatrics*.
